# Factors Influencing the Choice of Radiology Subspecialty Among Radiology Trainees in Saudi Arabia

**DOI:** 10.7759/cureus.6149

**Published:** 2019-11-13

**Authors:** Sultan T Alturki, Malek K Albusair, Fahad Alhumaid, Sayaf Alsharif, Khalid M Aljalajel, Faisal Aloufi, Abdulrahim Almotairy

**Affiliations:** 1 Radiology, King Fahad Medical City, Riyadh, SAU; 2 Emergency Medicine, College of Medicine, Al-Imam Mohammad Ibn Saud Islamic University, Riyadh, SAU; 3 Plastic Surgery, King Faisal Specialist Hospital and Research Centre, Jeddah, SAU; 4 Orthopedic, Prince Sultan Military Medical City, Riyadh, SAU; 5 Psychiatry, College of Medicine, Al-Imam Mohammad Ibn Saud Islamic University, Riyadh, SAU; 6 Radiology, King Faisal Specialist Hospital and Research Centre, Riyadh, SAU; 7 Radiology, Prince Mohammed Bin Abdulaziz Hospital, Riyadh, SAU

**Keywords:** training, residency, radiology, subspecialty, factors, saudi arabia, training

## Abstract

Background

Differential choices of radiology subspecialties by radiology trainees can cause shortages in some subspecialties. The objective of the current study was to evaluate the relative preference of different radiology subspecialties and the influencing factors among radiology trainees in Saudi Arabia.

Methods

An online questionnaire was developed based on previous publications and was used to collect the data from radiology trainees in Saudi Arabia during August 2018. The relative importance of potential personal and work-related factors was assessed using Likert-scaled responses.

Results

A total of 105 radiology trainees were included in the current analysis. Approximately 64.8% of the trainees were males. A total of eight subspecialties were reported, with the most frequent being interventional radiology (20%), neuroradiology (19%), abdominal/gastrointestinal (15.2%), and musculoskeletal (14.3%). Personal factors that were reported as extremely or very important included strong personal interest (84.8%), successful/enjoyable rotation during training (84.8%), and intellectual challenge (76.2%). Work-related factors that were reported as extremely or very important included direct impact on patient care (84.8%), advanced or a variety of imaging modalities (81%), direct professional contact (77.1%), and favorable/flexibility of working hours and on-call commitments (77.1%). The subspecialty of interventional radiology was more frequently chosen by male trainees (p = 0.006), while the gynecological/breast subspecialty was exclusively chosen by female trainees (p < 0.001).

Conclusion

In addition to gender-specific differences, we are reporting several important personal and professional factors that influence the choice of radiology subspecialty. These findings can potentially help the directors of radiology training in making evidence-based modifications to their residency programs to ensure the maintenance of a sufficient radiology workforce.

## Introduction

Radiology is a high-technology specialty that interfaces with nearly all surgical and medical disciplines [1]. Despite the fact that radiology has been for a long time one of the top competitive medical specialties, recent official reports in the United States (US) and United Kingdom (UK) showed an increasing mismatch between the number of applicants and the available residency positions in the last decade [2-3]. In Saudi Arabia, the radiology specialty is chosen by less than 2% of medical students, while general surgery, pediatrics, and internal medicine are top preferred specialties [4]. The decline in radiology preference is probably a multifactorial problem that involves awareness, economic factors, and lifestyle factors [5-7]. Additionally, some of these factors are gender-specific, adding to the complexity of the problem [8]. The decline in radiology preference can lead to shrinkage of the workforce and eventually, a critical shortage of radiologists [2, 9].

Maintaining a sufficient number of highly qualified radiologists in different healthcare settings is highly dependent on understanding the factors that influence the choice of radiology residency among medical students [10] and the factors that influence pursuing a career in a certain radiology subspecialty among radiology residents [11-14]. For the latter, differential choice of radiology subspecialties by radiology trainees caused or has been projected to cause shortages in some radiology subspecialties, such as pediatric radiology, women’s imaging, and nuclear medicine according to previous similar studies [12-13, 15-16]. The choice of radiology subspecialties has been linked to multiple factors, including both professional and personal factors [11-14]. In Saudi Arabia, there is a lack of data examining the factors influencing the choice of radiology subspecialties.

The objective of the current study was to evaluate the relative preference of different radiology subspecialties and the influencing factors among radiology trainees in Saudi Arabia.

## Materials and methods

Study design

This was an online survey study done among radiology trainees in Saudi Arabia. Ethical approval was obtained from the Ethical Review Committee Board of Al-Imam Mohammad Ibn Saud Islamic University, Riyadh, Saudi Arabia.

Instrument

An online questionnaire was developed based on a previous publication [11] and used to collect trainee information. The questionnaire included gender, subspecialty, current year of training, training hospital, and geographic region. Personal factors that could potentially influence the choice of radiology subspecialty included background prior to entering radiology, exclusion of subspecialties they did not like, the influence of an inspirational role model/mentor, intellectual challenge, strong personal interest, spousal\family considerations, and successful/enjoyable rotation during training. Work-related factors that could potentially influence the choice of radiology subspecialty included advanced or variety of imaging modalities, direct impact on patient care, favorable/flexibility of working hours and on-call commitments, patient contact, private work "income," practical "interventional" skills, research opportunities, teaching opportunities, and direct professional contact. The relative importance of potential personal and work-related factors was assessed using Likert-scaled responses: not important at all, slightly important, somewhat important, very important, and extremely important.

Recruitment

The questionnaire was uploaded to an online survey tool (SurveyMonkey.com) An online survey was sent to the trainees. Radiology trainees in different regions of Saudi Arabia were invited through email, WhatsApp messages, and social media (Facebook and Twitter). The online questionnaire was open during August 2018.

Statistical analysis

Data were presented as frequencies and percentages for categorical data and mean (M) and standard deviation (SD) for continuous data. Significant differences in the demographic characteristics in a specific subspecialty compared to all other subspecialties (combined) were evaluated using the Chi-square test or Fisher exact test (as appropriate). Personal and work-related scores were calculated by summing up individual scores, which was “1” for not important at all, “2” for slightly important, “3” for somewhat important, “4” for very important, and “5” for extremely important. Personal and work-related scores were then transformed to a 100-point scale for easy interpretation. Significant differences in the personal and work-related (median) scores were evaluated using the Wilcoxon signed-ranks test. All p-values were two-tailed. A p-value < 0.05 was considered significant. The IBM Statistical Package for Social Sciences (SPSS), v 25.0 (IBM SPSS Statistics, Armonk, NY) was used for statistical analysis.

## Results

A total of 105 radiology trainees were included in the current analysis. As shown in Table 1, approximately two-thirds (64.8%) of the trainees were males. The most frequently chosen subspecialty was interventional radiology (20%), followed by neuroradiology (19%), abdominal/gastrointestinal (15.2%), musculoskeletal (14.3%), cardiothoracic/chest (9.5%), nuclear medicine (9.5%), gynecological/breast (8.6%), and pediatrics (3.8%). The four years of training were fairly represented and ranged between 18.1% (Year 1) and 32.4% (Year 2). Approximately 2.9% of the trainees were fellows. The trainees were recruited from 18 hospitals in three Saudi regions. The majority of the trainees (77.1%) were trained in the central region, while 17.1% and 5.7% of the trainees (respectively) were trained in Western and Eastern regions.

**Table 1:** Demographic Data of Respondents R: residency year

	Number	Percentage
Gender		
Male	68	64.8%
Female	37	35.2%
Subspecialty		
Interventional Radiology	21	20.0%
Neuroradiology	20	19.0%
Abdominal/Gastrointestinal	16	15.2%
Musculoskeletal	15	14.3%
Cardiothoracic/Chest	10	9.5%
Nuclear Medicine	10	9.5%
Gynecological/Breast	9	8.6%
Pediatric	4	3.8%
Current Year of Training		
R1	19	18.1%
R2	34	32.4%
R3	28	26.7%
R4	21	20.0%
Fellow	3	2.9%
Geographic region		
Central region	81	77.1%
Western region	18	17.1%
Eastern region	6	5.7%

**Figure 1:**
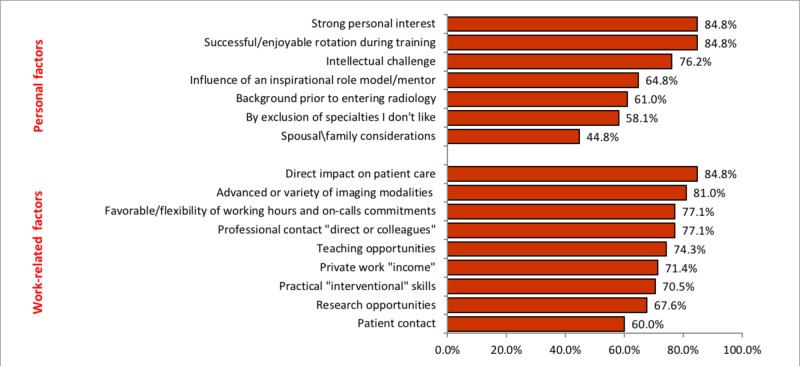
Extremely/very important personal and work-related factors that influence the choice of radiology subspecialty

The responses of trainees to the importance of different personal and work-related factors in influencing the choice of radiology subspecialty are shown in Table 2 and Figure 1. Personal factors that were reported as extremely or very important by more than 75% of the trainees included a strong personal interest (84.8%), successful/enjoyable rotation during training (84.8%), and intellectual challenge (76.2%). Work-related factors that were reported as extremely or very important by more than 75% of the trainees included direct impact on patient care (84.8%), advanced or variety of imaging modalities (81.0%), direct professional contact (77.1%), and favorable/flexibility of working hours and on-call commitments (77.1%). Personal and work-related factors that were reported as not important at all by at least 10% of the trainees included spousal\family considerations (23.8%), practical interventional skills (12.4%), research opportunities (10.5%), and patient contact (10.5%).

**Table 2:** Personal and Work-related Factors and Their Importance in Influencing the Choice of Radiology Subspecialty

	Not important at all	Slightly important	Somewhat important	Very important	Extremely important
Personal factors					
Background prior to entering radiology	10 (9.5%)	9 (8.6%)	22 (21.0%)	24 (22.9%)	40 (38.1%)
By exclusion of specialties I don't like	7 (6.7%)	6 (5.7%)	31 (29.5%)	27 (25.7%)	34 (32.4%)
Influence of an inspirational role model/mentor	3 (2.9%)	11 (10.5%)	23 (21.9%)	38 (36.2%)	30 (28.6%)
Intellectual challenge	2 (1.9%)	5 (4.8%)	18 (17.1%)	54 (51.4%)	26 (24.8%)
Strong personal interest	1 (1.0%)	2 (1.9%)	13 (12.4%)	34 (32.4%)	55 (52.4%)
Spousal\family considerations	25 (23.8%)	18 (17.1%)	15 (14.3%)	12 (11.4%)	35 (33.3%)
Successful/enjoyable rotation during training	3 (2.9%)	5 (4.8%)	8 (7.6%)	30 (28.6%)	59 (56.2%)
Work-related factors					
Advanced or variety of imaging modalities	1 (1.0%)	3 (2.9%)	16 (15.2%)	26 (24.8%)	59 (56.2%)
Direct impact on patient care	1 (1.0%)	5 (4.8%)	10 (9.5%)	23 (21.9%)	66 (62.9%)
Favorable/flexibility of working hours and on-call commitments	1 (1.0%)	5 (4.8%)	18 (17.1%)	35 (33.3%)	46 (43.8%)
Patient contact	11 (10.5%)	9 (8.6%)	22 (21.0%)	31 (29.5%)	32 (30.5%)
Private work "income"	4 (3.8%)	9 (8.6%)	17 (16.2%)	42 (40.0%)	33 (31.4%)
Practical "interventional" skills	13 (12.4%)	5 (4.8%)	13 (12.4%)	29 (27.6%)	45 (42.9%)
Research opportunities	11 (10.5%)	7 (6.7%)	16 (15.2%)	25 (23.8%)	46 (43.8%)
Teaching opportunities	2 (1.9%)	7 (6.7%)	18 (17.1%)	21 (20.0%)	57 (54.3%)
Professional contact "direct or colleagues"	3 (2.9%)	7 (6.7%)	14 (13.3%)	27 (25.7%)	54 (51.4%)

The associations between the choice of radiology subspecialty and the demographic and influencing factors are shown in Table 3. Interventional radiology subspecialty was more frequently chosen by male than female trainees (90.5% versus 9.5%, p = 0.006), while gynecological/breast subspecialty was exclusively chosen by female trainees (100.0% versus 0.0%, p < 0.001). Gynecological/breast subspecialty was more frequently chosen by senior (R ≥ 3) than junior (≤ R2) female trainees (44.4% in R3 and 55.6% in R4 versus 0.0% in ≤ R2, p = 0.008). There were no significant associations between the choice of radiology subspecialty and the geographic region of the training center (p > 0.05 in all subspecialties). The overall work-related score (81% ± 14%) was slightly higher than the overall personal score (77% ± 15%); the difference was statistically significant (p = 0.003). This was clearly shown in neuroradiology (81% ± 14% versus 73% ± 14%, p = 0.023) and to a lesser extent, in gynecological/breast (86% ± 12% versus 70% ± 14%, p = 0.086).

**Table 3:** Associations Between the Choice of Radiology Subspecialty and the Demographic and Influencing Factors * Number and percentage ** mean ± standard deviation p-value^1^: examines the difference between relevant subspecialty and all other subspecialties together p-value^2^: examines the difference between personal and work-related factors in the relevant subspecialty

	Interventional Radiology	Neuro-radiology	Abdominal /gastrointestinal	Musculo-skeletal	Cardiothoracic /chest	Nuclear medicine	Gynecological/ breast	Pediatric
	N = 21	N = 20	N = 16	N = 15	N = 10	N = 10	N = 9	N = 4
Gender*								
Male	19 (90.5%)	16 (80.0%)	12 (75.0%)	9 (60.0%)	6 (60.0%)	5 (50.0%)	0 (0.0%)	1 (25.0%)
Female	2 (9.5%)	4 (20.0%)	4 (25.0%)	6 (40.0%)	4 (40.0%)	5 (50.0%)	9 (100.0%)	3 (75.0%)
P-value^1^	0.006	0.113	0.352	0.677	0.739	0.318	< 0.001	0.124
Current year of training*								
R1	7 (33.3%)	5 (25.0%)	1 (6.3%)	2 (13.3%)	1 (10.0%)	2 (20.0%)	0 (0.0%)	1 (25.0%)
R2	9 (42.9%)	4 (20.0%)	6 (37.5%)	6 (40.0%)	4 (40.0%)	4 (40.0%)	0 (0.0%)	1 (25.0%)
R3	3 (14.3%)	5 (25.0%)	3 (18.8%)	6 (40.0%)	3 (30.0%)	3 (30.0%)	4 (44.4%)	1 (25.0%)
R4	2 (9.5%)	4 (20.0%)	5 (31.3%)	1 (6.7%)	2 (20.0%)	1 (10.0%)	5 (55.6%)	1 (25.0%)
Fellow	0 (0.0%)	2 (10.0%)	1 (6.3%)	0 (0.0%)	0 (0.0%)	0 (0.0%)	0 (0.0%)	0 (0.0%)
P-value^1^	0.112	0.189	0.322	0.488	0.954	0.933	0.008	> 0.99
Geographic region*								
Central region	19 (90.5%)	15 (75.0%)	13 (81.3%)	10 (66.7%)	7 (70.0%)	8 (80.0%)	6 (66.7%)	3 (75.0%)
Western region	2 (9.5%)	5 (25.0%)	3 (18.8%)	3 (20.0%)	1 (10.0%)	2 (20.0%)	2 (22.2%)	0 (0.0%)
Eastern region	0 (0.0%)	0 (0.0%)	0 (0.0%)	2 (13.3%)	2 (20.0%)	0 (0.0%)	1 (11.1%)	1 (25.0%)
P-value^1^	0.303	0.326	0.779	0.259	0.128	> 0.99	0.505	0.331
Overall scores of influencing factors**								
Personal	83% ± 13%	73% ± 14%	70% ± 13%	79% ± 17%	76% ± 15%	82% ± 16%	70% ± 14%	87% ± 11%
Work-related	85% ± 10%	81% ± 14%	72% ± 13%	80% ± 16%	80% ± 11%	81% ± 19%	86% ± 12%	83% ± 16%
Overall	84% ± 10%	78% ± 12%	71% ± 12%	80% ± 15%	78% ± 12%	82% ± 17%	79% ± 08%	85% ± 10%
P-value^2^	0.297	0.023	0.099	0.975	0.241	0.677	0.086	0.715

The relative importance of individual influencing factors by the choice of each radiology subspecialty is shown in Table 4. For example, the top influencing factors among those who chose interventional radiology included gaining practical "interventional" skills (93% ± 10%), a direct impact on patient care (91% ± 16%), and strong personal interest (89% ± 16%).

**Table 4:** Relative Importance* of Individual Influencing Factors by the Choice of Radiology Subspecialty

	Interventional Radiology	Neuro-radiology	Abdominal /gastrointestinal	Musculo-skeletal	Cardiothoracic /chest	Others
	N = 21	N = 20	N = 16	N = 15	N = 10	N = 23
Personal factors						
Background prior to entering radiology	88% ± 16%	73% ± 24%	63% ± 30%	71% ± 29%	56% ± 35%	82% ± 20%
By exclusion of specialties I don't like	77% ± 22%	67% ± 28%	64% ± 23%	79% ± 27%	78% ± 20%	81% ± 18%
Influence of an inspirational role model/mentor	83% ± 17%	69% ± 21%	78% ± 25%	71% ± 24%	78% ± 20%	75% ± 21%
Intellectual challenge	82% ± 09%	79% ± 18%	74% ± 19%	76% ± 25%	80% ± 16%	79% ± 19%
Strong personal interest	89% ± 16%	88% ± 18%	79% ± 17%	93% ± 10%	88% ± 14%	84% ± 20%
Spousal\family considerations	74% ± 28%	54% ± 33%	46% ± 30%	75% ± 32%	66% ± 34%	62% ± 32%
Successful/enjoyable rotation during training	88% ± 22%	83% ± 20%	85% ± 16%	91% ± 13%	84% ± 25%	86% ± 24%
Work-related factors						
Advanced or variety of imaging modalities	89% ± 15%	94% ± 11%	83% ± 22%	85% ± 16%	72% ± 27%	88% ± 17%
Direct impact on patient care	91% ± 16%	87% ± 21%	88% ± 23%	83% ± 20%	92% ± 10%	89% ± 17%
Favorable/flexibility of working hours and on-call commitments	81% ± 17%	84% ± 18%	73% ± 25%	83% ± 17%	94% ± 10%	86% ± 18%
Patient contact	76% ± 19%	65% ± 30%	64% ± 28%	72% ± 25%	74% ± 27%	80% ± 26%
Private work "income"	81% ± 18%	81% ± 19%	66% ± 25%	80% ± 25%	74% ± 21%	78% ± 21%
Practical "interventional" skills	93% ± 10%	65% ± 33%	60% ± 26%	76% ± 32%	82% ± 18%	82% ± 24%
Research opportunities	88% ± 18%	80% ± 27%	60% ± 27%	76% ± 29%	68% ± 34%	80% ± 24%
Teaching opportunities	85% ± 24%	86% ± 21%	77% ± 23%	85% ± 26%	86% ± 17%	83% ± 18%
Professional contact "direct or colleagues"	84% ± 24%	87% ± 16%	76% ± 25%	84% ± 25%	80% ± 19%	85% ± 20%

## Discussion

We are reporting the preference rate of different radiology subspecialties and the factors influencing such preference among a sample of radiology trainees recruited from 18 hospitals in three Saudi regions. Top personal influencing factors in our trainees included strong personal interest, successful/enjoyable rotation during training, and intellectual challenge. Similarly, these three factors were the top personal influencing factors among UK radiology trainees who answered in an exactly similar survey tool [11]. Additionally, strong personal interest and intellectual challenge were the top personal influencing factors among US radiology trainees who answered a very close survey tool [15]. On the other hand, top work-related influencing factors in our trainees included the direct impact on patient care and availability of advanced or variety of imaging modalities. Similarly, these two factors were the top work-related influencing factors among UK radiology trainees [11], while the availability of advanced or a variety of imaging modalities was the first work-related influencing factor among US radiology trainees [15]. Interestingly, using a different tool, personal interest remained the first personal factor while enhanced employability was the first work-related factor reported by Canadian radiology residents planning to purse fellowship training [12].

Interventional radiology and neuroradiology were the top chosen subspecialties among our trainees. This was similar to reports from the US, UK, and Canada where both specialties were among the top four choices [11, 13, 15-16]. However, the choice of these subspecialties showed some fluctuations over the last 10 - 15 years, probably reflecting the job market. For example, the trend of choosing interventional radiology in the US showed some decrease over time [16]. Additionally, the choice of neuroradiology in Canada has moved towards the bottom in recent years [12]. Moreover, not all those who plan interventional radiology early in the training actually do continue the same choice by the end of residency training [17]. The decreased interest in interventional radiology has been attributed to stressful work experience with increased radiation exposure and an undesirable lifestyle [13]. On the other hand, the choice of interventional radiology in our trainees was largely influenced by gaining practical "interventional" skills, a direct impact on patient care, and strong personal interest. The choice of neuroradiology in our trainees was largely influenced by the availability of advanced or variety of imaging modalities, direct impact on patient care, and strong personal interest.

Pediatric radiology, women’s imaging, and nuclear medicine were the least chosen subspecialties among our trainees. Similarly, these three subspecialties have been consistently reported in the bottom of the choice list in Western countries [12-13, 15-16]. It has been suggested that the reluctance of choosing pediatric radiology may be caused by a limited job market for pediatric radiology which is practiced mainly in major academic centers, thus depriving the applicants of private work and a better salary [13, 15]. Additionally, nuclear medicine is perceived as a too stressful subspecialty, while mammography is perceived as not an interesting field [13]. The choice of pediatric radiology, women’s imaging, and nuclear medicine in our trainees were largely influenced by direct impact on patient care, the availability of advanced or a variety of imaging modalities, successful/enjoyable rotation during training, and favorable/flexibility of working hours and on-call commitments.

Females represented approximately one-third of our sample of radiology trainees. The under-presentation of females in the radiology specialty is well-known. For example, only one-fourth of radiology residents in the US and Canada are females despite the fact that they represent approximately half of the medical student graduates [8, 18]. While there is no conclusive explanation of such under-presentation [19], it may be related to the fear of radiation risk. Interestingly, the current finding showed gender-specific differences in the choice of radiology subspecialties, with a male predominance in interventional radiology and female exclusiveness in the gynecological/breast subspecialty. It has been reported that female radiologists are usually clustered in certain subspecialties, such as mammography and sonography, while avoiding interventional and vascular radiology [19]. The reluctance of our male trainees to choose gynecological/breast radiology may reflect the very conservative society in Saudi Arabia where female patients prefer to deal with a female doctor.

## Conclusions

The current study is the first study to examine the factors influencing the choice of different radiology subspecialties among radiology trainees in Saudi Arabia. The trainees were recruited from three Saudi regions and the list of factors included 16 different personal and professional factors. Additionally, gender-specific differences in preference were discussed. However, being a convenience sample, the current findings should be generalized with caution. Additionally, the relatively small sample size may have masked some of the associations between the studied factors and the choice of subspecialties. Nevertheless, the finding is considered a unique addition to the field that can potentially help the directors of radiology training in making evidence-based modifications to the residency programs to ensure the maintenance of a sufficient radiology workforce.
